# Supplementary material for: Iterative delay correction improves breath-hold cerebrovascular reactivity mapping in clinical populations
Source: Imaging Neurosci (Camb). 2026 Oct 1;4:IMAG.a.1386. doi: 10.1162/IMAG.a.1386 (PMC13636296; doi:10.1162/IMAG.a.1386)
Supplement: Supplementary file 1 [file IMAG.a.1386_supp.pdf]

Iterative delay correction improves breath-hold cerebrovascular reactivity mapping in clinical populations

Rebecca G. Clements, Fatemeh Geranmayeh, Niamh V. Parkinson, Molly G. Bright

Supplementary Material

Validation of the iterative approach using simulated datasets:

- Ground-truth voxel-wise CVR amplitude and delay maps were created using maps from a participant with stroke. Because this participant did not exhibit a region with markedly prolonged delays, a lesion mask was manually drawn (see white outline) and delay values within the mask were assigned. To better understand the utility of the iterative approach for different ranges of extreme delay values, we ran two different simulations. For Simulation #1 (Figure S1), the voxels in the simulated lesion mask were randomly assigned delay values ranging from 13 to 16 seconds in 0.5 second increments. For Simulation #2 (Figure S2), the voxels in the simulated lesion mask were randomly assigned delay values ranging from 16.5 to 19 seconds in 0.5 seconds increments.
- For each set of ground truth CVR amplitude and delay maps, we used a high-quality $P_{ET}CO_2$ trace to generate simulated fMRI data. For each voxel, the $P_{ET}CO_2$ trace was shifted according to the voxel's ground-truth delay and scaled by its ground-truth CVR amplitude to create the expected BOLD signal. A baseline intensity of 1000 was added, and Gaussian noise was introduced with a standard deviation equal to 25% of the signal standard deviation. Thus, 2 simulated fMRI datasets were generated from known CVR amplitude and delay maps, allowing us to directly evaluate how accurately each method recovered the ground-truth values.
- CVR amplitude and delay maps were estimated from the simulated data using the $P_{ET}CO_2$ regressor and compared with the known ground-truth maps.
- For Simulation #1 (Figure S1), we found that using a 9 second maximum delay resulted in delay values being underestimated, particularly in the simulated lesion. Compared to the 9 second maximum-delay approach (second row), the iterative method produced lower mean absolute error (MAE) of CVR delay maps at both the whole-brain and lesion level (third row). The iterative approach also resulted in lower MAE of CVR amplitude in the simulated lesion.
- For Simulation #2 (Figure S2), using a 9 second maximum delay resulted in delay values in the simulated lesion being incorrectly calculated as early rather than late, and amplitude values in the simulated lesion being incorrectly calculated as negative rather than positive. The iterative method resulted in the

delay values being correctly calculated as late and amplitude values being correctly calculated as positive. Again, the iterative approach produced lower MAE of CVR delay at both the whole brain and lesion level, and lower MAE of CVR amplitude at the lesion level.

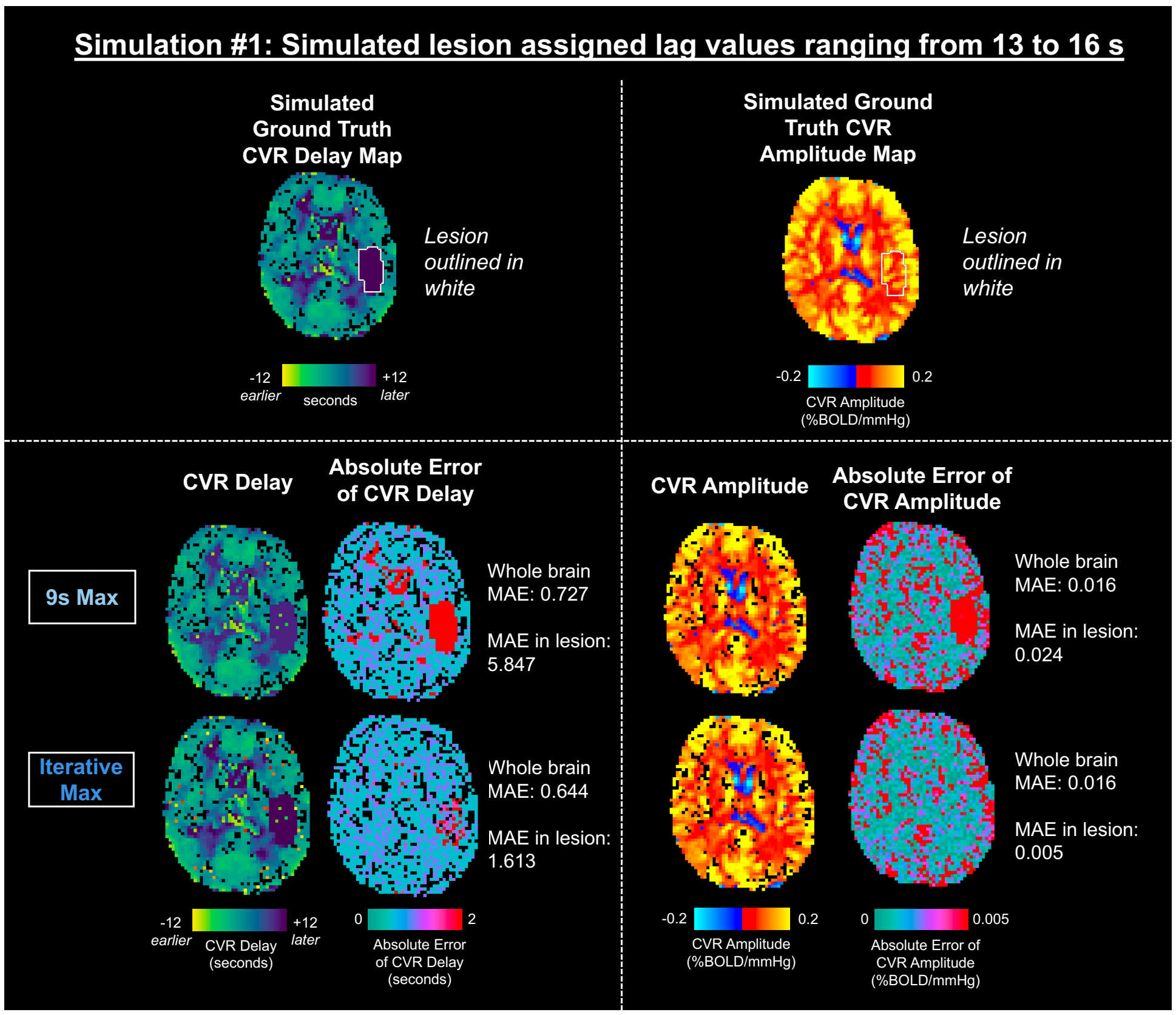

Figure S1. Validation of the iterative approach using Simulated Dataset #1. Ground-truth, voxel-wise CVR amplitude and delay maps were created using maps from a participant with stroke (first row). A lesion mask was manually drawn (see white outline) and assigned delay values within the mask ranging from 13 to 16 s in 0.5 s increments to generate a ground-truth delay map with an area of extreme delay. Using a 9 second maximum resulted in underestimation of delay values, particularly in the simulated lesion. Compared to the 9-second maximum-delay approach, the iterative approach produced lower MAE of CVR delay at both the whole-brain (0.644 seconds compared to 0.727 seconds) and lesion (1.613 seconds compared to 5.847 seconds) level. The iterative approach also produced lower MAE of CVR amplitude within the simulated lesion region (0.005 %BOLD/mmHg compared to 0.024 %BOLD/mmHg).

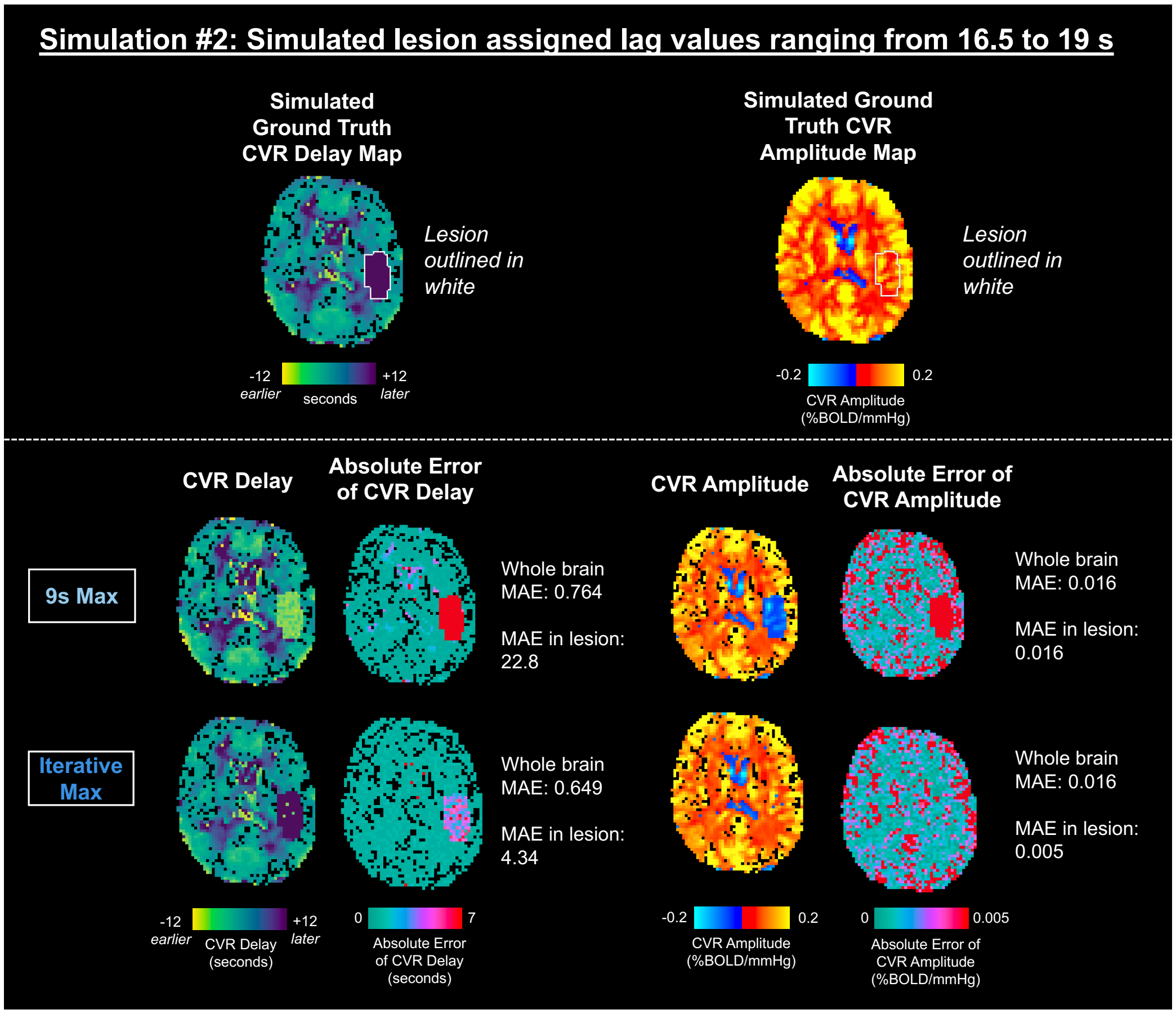

Figure S2. Validation of the iterative approach using Simulated Dataset #2. Ground-truth, voxel-wise CVR amplitude and delay maps were created using maps from a participant with stroke (first row). A lesion mask was manually drawn (see white outline) and assigned delay values within the mask ranging from 16.5 to 19 s in 0.5 s increments to generate a ground-truth delay map with an area of extreme delay. Using a 9 second maximum resulted in the polarity of CVR amplitude and delay being incorrectly calculated as negative and positive, respectively, in the simulated lesion. The iterative approach resulted in CVR amplitude and delay estimates having the correct polarity. Compared with the 9-second maximum-delay approach, the iterative approach produced lower MAE of CVR delay at both the whole-brain (0.649 seconds compared to 0.764 seconds) and lesion (4.34 seconds compared to 22.8 seconds) level. The iterative approach also produced lower MAE of CVR amplitude within the simulated lesion region (0.005 %BOLD/mmHg compared to 0.016 %BOLD/mmHg).

Table S1. Percentage of ROIs that became significant with an iterative maximum

ROI	Median % of ROI that became significant with an iterative maximum

	Voxels initially at the late response (+9s) boundary (median ± SD)	Voxels initially at the early response (-9s) boundary (median ± SD)
Normal appearing GM	0.14 ± 0.6	0.08 ± 0.4
Normal appearing WM	0.19 ± 0.5	0.11 ± 0.4
CSF	0.21 ± 0.5	0.10 ± 0.4
Ventricles	0.47 ± 0.9	0.21 ± 0.4
Periventricular space	0.22 ± 0.5	0.19 ± 0.4
Stroke lesions	0.11 ± 3	0 ± 0.7
White matter hyperintensities (WMHs)	0.24 ± 0.9	0 ± 1
Combined mask of stroke lesions and WMHs (and lacunes if present)	0.50 ± 0.5	0.32 ± 0.8

Note regarding Table S1: It appears that when lesions and WMHs are analyzed separately, the median percentage of voxels that become significant is much lower (and with a larger standard deviation) than when they're combined into a single "vascular lesion" mask. This seems to reflect the fact that in this group with relatively small lesions and WMHs (stroke lesions and WMHs occupied a median of 0.25% and 0.27% of all brain voxels, respectively), the iterative maximum produces only a small number of voxels that change significance per subject, and in this dataset, these voxels are not always consistently localized to either lesions or WMHs. By separating lesions and WMHs, the voxels that switch to being significant are more likely to be diluted by zero-valued observations across participants. In contrast, combining the two masks leads to a more stable estimate of the median percentage across subjects. Future work is needed in a larger sample size and in participants with larger lesion loads to better

understand the spatial location of voxels that become significant with the iterative approach. Additionally, future work examining other brain regions such as microbleeds would be valuable for a more comprehensive understanding of these effects.

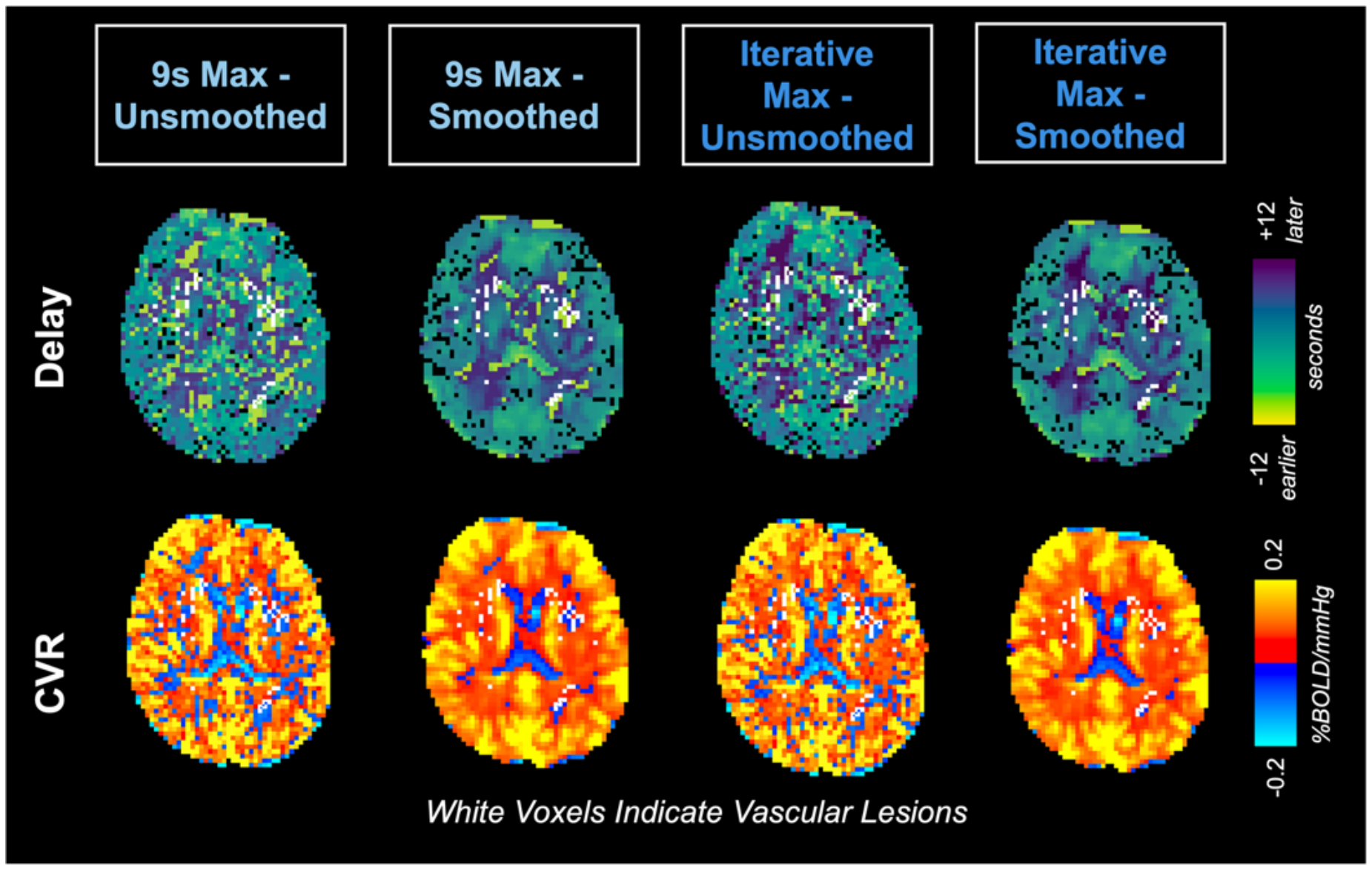

Figure S3. CVR maps for one example subject generated with and without spatial smoothing. Vascular lesions are outlined in white. Although the smoothed maps appear less noisy, the iterative delay correction approach produces the same overall effect with and without smoothing. Specifically, compared with the maps generated using a 9 second maximum delay, the iterative approach results in increased delay estimates and regions where CVR values switch from negative to positive. This effect is discussed in more detail in Section 3.1, where this subject corresponds to Example 2 in Figure 5.

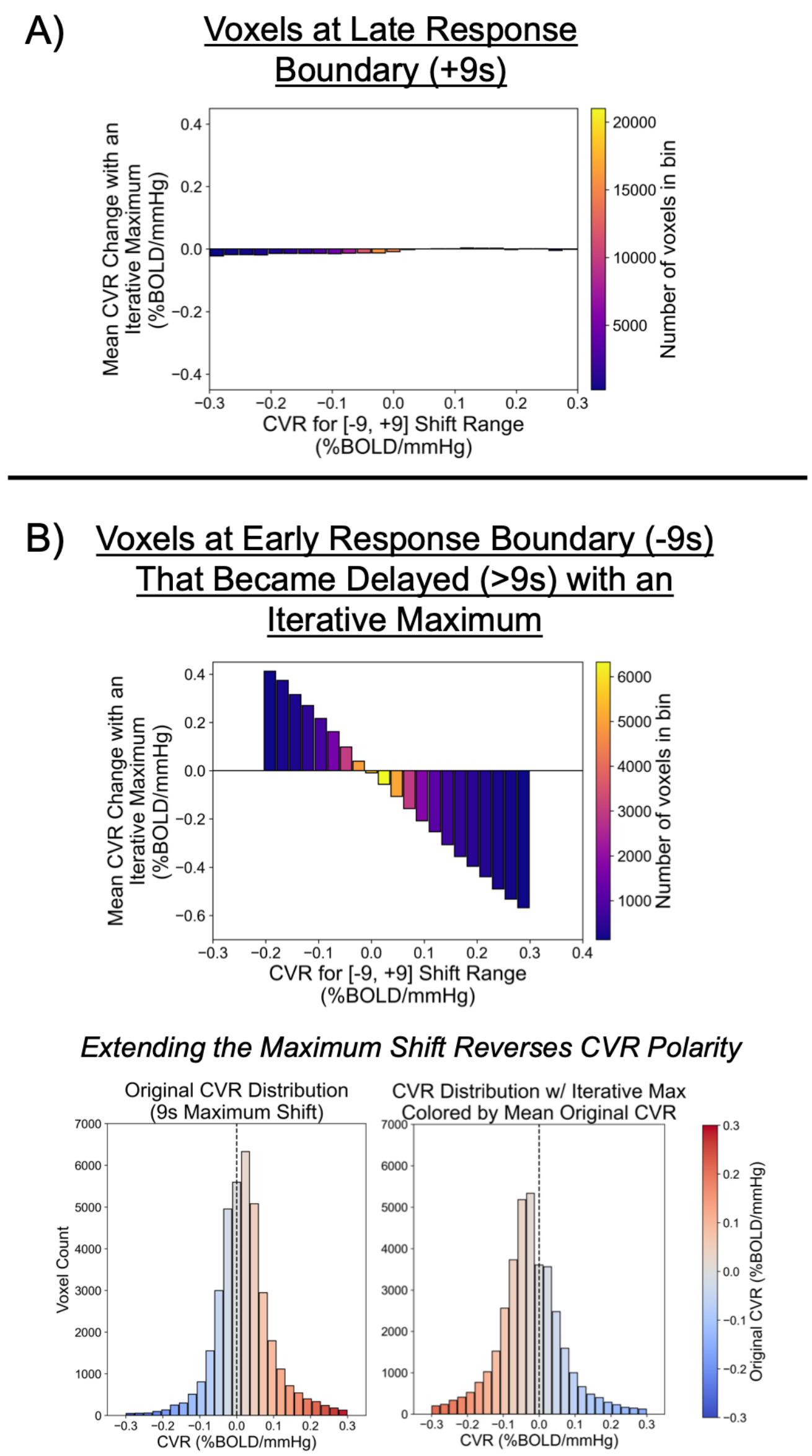

Figure S4. Changes in CVR amplitude with an iterative maximum shift for voxels at the late response boundary (A) and the early response boundary that became delayed with an iterative maximum (B). This figure is the same as Figure 4 in the main text, except that it includes all voxels, rather than voxels with statistically significant CVR. The main conclusions from Figure 5 remain true for this figure.
